# The Role of Lectin-Carbohydrate Interactions in the Regulation of ER-Associated Protein Degradation

**DOI:** 10.3390/molecules20069816

**Published:** 2015-05-27

**Authors:** Monika Słomińska-Wojewódzka, Kirsten Sandvig

**Affiliations:** 1 Department of Molecular Biology, University of Gdańsk, Wita Stwosza 59, 80-308 Gdańsk, Poland; 2 Department of Molecular Cell Biology, Institute for Cancer Research, Oslo University Hospital, The Norwegian Radium Hospital, 0379 Oslo, Norway; E-Mail: kirsten.sandvig@ibv.uio.no; 3 Centre for Cancer Biomedicine, Faculty of Medicine, University of Oslo, 0379 Oslo, Norway; 4 Department of Biosciences, University of Oslo, 0316 Oslo, Norway

**Keywords:** *N-*glycan, EDEM chaperone proteins, ERAD

## Abstract

Proteins entering the secretory pathway are translocated across the endoplasmic reticulum (ER) membrane in an unfolded form. In the ER they are restricted to a quality control system that ensures correct folding or eventual degradation of improperly folded polypeptides. Mannose trimming of *N-*glycans on newly synthesized proteins plays an important role in the recognition and sorting of terminally misfolded glycoproteins for ER-associated protein degradation (ERAD). In this process misfolded proteins are retrotranslocated into the cytosol, polyubiquitinated, and eventually degraded by the proteasome. The mechanism by which misfolded glycoproteins are recognized and recruited to the degradation machinery has been extensively studied during last decade. In this review, we focus on ER degradation-enhancing α-mannosidase-like protein (EDEM) family proteins that seem to play a key role in the discrimination between proteins undergoing a folding process and terminally misfolded proteins directed for degradation. We describe interactions of EDEM proteins with other components of the ERAD machinery, as well as with various protein substrates. Carbohydrate-dependent interactions together with *N-*glycan-independent interactions seem to regulate the complex process of protein recognition and direction for proteosomal degradation.

## 1. Introduction

The endoplasmic reticulum (ER) is an essential cellular compartment for protein synthesis and maturation. Nearly one-third of all newly synthesized proteins in the human cells are targeted to the ER, which is the first step in the delivery of these proteins for trafficking to other organelles of the secretory pathway, the plasma membrane or to the extracellular space [1,2]. The milieu of the ER differs from that of the cytosol with respect to ions and redox conditions. Some co-translational and post-translational protein modifications, e.g., disulphide bonds formation, specific proteolytic cleavages, initial steps in *N*-glycosylation or glycophosphatidylinositol (GPI)-anchor addition take place exclusively in the rough ER. The majority of proteins synthesized in the ER are glycoproteins. Addition and processing of carbohydrates of these proteins serve highly diverse functions. They stabilize the proteins against denaturation and proteolysis, enhance solubility, facilitate orientation of proteins relative to a membrane, confer structural rigidity to proteins, and attune the charge and isoelectric point of proteins (for a review see [3]). Moreover, *N-*linked oligosaccharide moieties serve as ligands in a variety of recognition processes. They modulate immune response, mediate interactions with pathogens, and regulate protein turnover. The developmental importance of *N-*glycosylation is reflected in such functions as morphogenesis, proliferation, differentiation, and apoptosis [4]. No other covalent protein modification is as common and as complex chemically, and no other modification is employed for so many different purposes.

ER contains high concentrations of dedicated molecular chaperones, folding enzymes and quality control factors that facilitate correct folding of newly synthesized polypeptides and ensure that only properly folded and assembled proteins are transported to their final destination through the secretory pathway [5]. Unfolded, misfolded, or partly folded and assembled proteins are selectively retained in the ER. They become recognized as aberrant products and retrotranslocated to the cytosol for 26S proteasome degradation in a series of tightly regulated processes called ER-associated degradation (ERAD) [6,7]. In this review we summarize recent data highlighting the involvement of *N-*glycans in protein folding and in the regulation of glycoprotein degradation by ERAD. These events involve a network of folding sensors, glycosyltransferases, and glycosidases. We will focus especially on lectins and their functions in the protein turnover. Lectins can recognize various protein substrates also in a glycan independent manner, what significantly contributes to our understanding of ERAD.

## 2. The Role of Carbohydrates in the ER Protein Folding

### 2.1. N-Linked Glycans Core Formation

**Figure 1:**
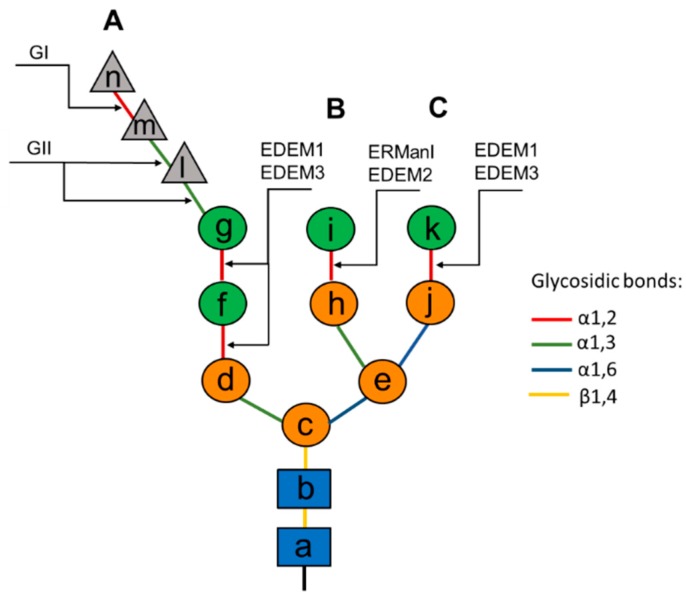
Structure of *N-*linked oligosaccharides. The pre-formed oligosaccharide covalently attached to Asn-Xxx-Ser/Thr sequences of nascent polypeptide chains is composed of three glucoses (grey triangles), nine mannoses (green and orange circles) and two *N-*acetylglucosamines (blue squares). Mannose residues that are removed by members of the glycosyl hydrolase family 47 (ER mannosidase I (ERManI) or EDEM1, EDEM2, EDEM3) are marked as green circles. It should be noted that extensive trimming of these mannoses by ERManI and/or Golgi endomannosidases was also reported (see text). Glucose n is removed by glucosidase I (GI), glucose residues m and l are both removed by glucosidase II (GII). A, B and C define the oligosaccharide branch. Letters a–n specify particular sugar residues. These letters are used throughout the text. The type of glycosidic bond is shown in color.

Nascent polypeptide chains enter the ER lumen through the ER membrane protein channel formed by Sec61αβγ translocon complex. The asparagine residue of a consensus motif (Asn-Xxx-Ser/Thr or more rarely Asn-Xxx-Cys, Asn-Xxx-Val, or Asn-Gly) is rapidly modified through the covalent attachment of a pre-formed oligosaccharide core that is comprised of two *N-*acetyl glucosamines, nine mannoses and three glucoses (Glc_3_Man_9_GlcNAc_2_) [8] (Figure 1). The transfer generally occurs co-translationally once the consensus sequence has emerged with 12–14 amino acids (30–40 Å) into the ER lumen aligning the Asn with the active site of the oligosaccharyl transferase (OST), a multisubunit enzyme that transfers preassembled glycans to the Asn residue [9]. 

Glycosylation efficiency depends on many cellular factors such as the translation rate, the level of OST, and the availability of the pre-formed oligosaccharide core bound to the ER membrane lipid donor dolichol pyrophosphate. It has been proposed that defined subunits of the OST might act as chaperones or enzymes to modulate, or even prevent, the folding of the target proteins in order to facilitate *N-*glycosylation [10]. As a result of this complex cellular balance two-thirds of available potential *N-*glycosylation sites can be occupied [11]. These sites can occur in all forms of secondary structure, with a bias toward turns and bends. However, there is a highly increased probability of glycosylation sites occurring at or just after points in the chain where there is a change in the secondary structure. This raises the possibility that glycosylation favours reorientation of the peptide chain [11]. The localization of glycans is at least partially limited to flexible regions since there is a requirement in the transfer reaction for the hydroxyl group of the Ser/Thr residue in the consensus site to loop around and increase the nucleophilic properties of the relatively chemically unreactive Asn residue [12,13]. *N-*glycans are hydrophilic structures that extend for about 30 Å from the protein backbone [14]. This feature efficiently prevents aggregation of the yet unstructured nascent chains. They rapidly become accessible to ER-resident sugar-processing enzymes and their subsequent modification determines the fate of the associated polypeptide chain.

### 2.2. Glycan-Dependent Folding in the Lectins Calnexin-Calreticulin Chaperone System 

**Figure 2:**
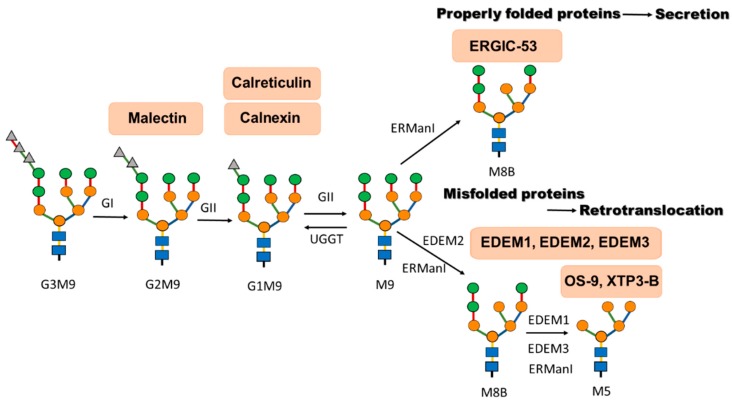
Glycan processing determines the fate of folding-competent and folding-defective glycoproteins. Nascent chains are glycosylated by addition of the 14 subunit oligosaccharide core (see Figure 1). After cleavage of the first glucose residue, di-glucosylated polypeptides (G2M9) associate with malectin. α-glucosidase II (GII) removes the second glucose, generating mono-glucosylated polypeptides (G1M9), which enter calnexin/calreticulin cycle. Once released from calnexin or calreticulin, glycoproteins are deglucosylated (M9) by glucosidase II. Re-glucosylation by UDP-glucose:glycoprotein glucosyltransferase (UGGT) enables the re-association of the polypeptides with calnexin or calreticulin. ER mannosidase I (ERManI) removes one mannose residue, generating eight mannose residues polypeptide (M8B). Correctly folded polypeptides are exported from the ER. Exit may be assisted by mannose lectins, such as ERGIC-53. Terminally misfolded proteins are further processed by EDEM family lectins. It is possible that aberrantly misfolded proteins are recognized by ERManI and/or EDEM2 in order to generate eight mannose residues polypeptides. Then EDEM1 together with EDEM3 (and/or ERManI) cleave three more mannose residues. Extensively demannosylated polypeptides (M5) can recruit lectins OS-9 and XTP3-B. However, the removal of mannose g is not a prerequisite for binding to OS-9. EDEMs can probably play dual role in ERAD: by acting directly or indirectly as mannosidases and by directing (chaperoning) misfolded proteins for degradation.

The triglucosylated form of the protein-bound oligosaccharide has a half-life of a few seconds. Thus, glycan processing starts immediately after its transfer from a dolichol pyrophosphate derivative to Asn residues in nascent polypeptide chains [3]. Glycan processing begins from the cleavage of the outermost glucose residue (glucose n, Figure 1) by α-glucosidase I (GI). This α-1,2-exoglucosidase is a type II membrane protein member of the glycosyl hydrolase (GH) family 63 [15]. The rapid GI-mediated deglucosylation of the protein-linked glycan, as well as the apparent inability of the enzyme to remove *in vivo* (but not *in vitro*) the glucose from the dolichol-P-P-linked glycan, strongly suggests the existence of a specific complex formed by the oligosaccharyltransferase, GI, and the dolichol derivative, with a very precise orientation of the components [16]. GI generates the ligand for a membrane-bound, ER lectin called malectin [17,18] (Figure 2). 

This recently discovered protein is induced under ER stress and is proposed to preferentially associate with immature and/or misfolded proteins to retain them in the ER. The capacity of malectin to detect terminally misfolded proteins so early after their expression in the ER lumen provides important biological significance for the di-glucosylated forms of protein-bound oligosaccharides. Further processing of the *N-*glycan includes removal of glucose m by α-glucosidase II (GII) (Figure 1). This α1,3 exoglucosidase is a luminal member of the GH family 31 and contains a catalytic α-subunit and a regulatory β-subunit [15]. Monoglucosylated glycan forms are then recognized by two ER resident lectins, calnexin (cnx) and calreticulin (crt), initially named for their ability to bind calcium [19,20] (Figure 2). Calnexin is a type I membrane protein, calreticulin is a soluble paralog of calnexin possessing 39% sequence homology [20,21,22,23]. Both lectins contain a single *N-*terminal globular carbohydrate binding domain and a second domain termed the P-domain named for its richness in proline residues. The P-domain is shaped in an overall hairpin like structures that in calnexin extends 140 Å away from the lectin binding domain [24]. In calreticulin this domain is shorter and with different construction of proline rich motifs [25], for review see also [26]. The P domain recruits an accessory oxidoreductase ERp57 involved in disulfide bond formation and isomerization, a rate-limiting step of protein folding in the ER [27,28,29]. 

Binding to calnexin and calreticulin can start co-translationally if the first monoglucosylated oligosaccharide is located within 50 residues of the polypeptide *N-*terminal end [30]. The number of cellular calnexin and calreticulin substrates is unknown, but it is considered that all newly synthetized *N-*glycosylated proteins can associate with one or both lectin chaperones. However, it should be noted that despite similarities between calnexin and calreticulin, striking differences have been observed in their specific substrate binding, what is reflected by the variations in the location and the number of glycans recognized [31,32,33,34]. Upon glycoprotein release from calnexin/calreticulin cycle, glucosidase II removes the final glucose creating the unglucosylated substrate (Figure 2). This step inhibits substrate rebinding to the lectin chaperones. Properly folded polypeptides are transported along the secretory pathway, whereas unfolded proteins are retained in the ER and can again be recruited to the calnexin/calreticulin cycle (Figure 2). 

Generally, glycoproteins can fold properly after a single association with a lectin chaperone, as was observed in mammalian cells [35] and in *Schizosaccharomyces pombe* [36]. However, it has been demonstrated that repeated cycles of association with and dissociation from calnexin/calreticulin cycle might play an important role in the proper maturation of at least some glycoproteins [37,38]. Regeneration of the monoglucosylated state and rebinding to the lectin chaperones calnexin and calreticulin is controlled by the UDP-glucose:glycoprotein glucosyltransferase (UGT1 or UGGT), a member of the glycosyltransferase family 24 [15] (Figure 2). UGGT consists of a large *N-*terminal domain responsible for the selection of protein substrates and a C-terminal carbohydrate transferase domain [39]. It has recently been demonstrated that *N-*terminal region of UGGT contains three tandem thioredoxin (trx)-like domains [40]. These domains are common to members of the protein disulfide isomerase (PDI) family, which are responsible for correct disulfide bond formation of ER proteins. None of the Trx-like domains of UGGT possess the CXXC catalytic motif, indicating that this enzyme is not directly involved in thiol/disulfide exchange reactions. However, it was demonstateted that noncatalytic Trx-like domains might be involved in substrate recognition [40,41,42]. UGGT is able to recognize polypeptides possessing deglucosylated oligosaccharides and to add glucose l, which results in re-glucosylated mannose g (Figure 1 and Figure 2). Importantly, the UGGT has striking substrate specificity: it prefers near-native molten globule-like folding intermediates and orphan subunits, and ignores native or extensively misfolded proteins [43,44,45]. How this specificity is accomplished remains unclear. Nevertheless, UGGT can unquestionably be considered as a crucial ER folding sensor that provides the essential connection between recognition of non-native polypeptide structures and oligosaccharide modifications responsible for recruiting chaperones.

## 3. Recognition and Processing of *N*-Glycan Structures during ER-Associated Degradation (ERAD)

### 3.1. Demannosylation of Misfolded Glycoproteins

It is not fully understood how the re-glucosylation/deglucosylation cycles are terminated. It is considered that a certain time frame is given for proper folding of each glycoprotein. If this folding time is exceeded and the glycoprotein molecule cannot achieve its proper conformation, it is finally targeted for ERAD. Among ERAD substrates are also extensively misfolded polypeptides that passed the calnexin/calreticulin cycle only once and were not recognized by UGGT. Signals and mechanisms that regulate recognition of folding-defective polypeptides expressed in the ER have been intensively studied for more than a decade, most extensively in yeast *Saccharomyces cerevisiae*; however recent experiments in mammalian cells have revealed the conservation of this process and majority of its components. 

Accumulating evidence indicates the crucial role of α1,2-mannosidase(s) in the generation of carbohydrate degradation signals [3,46,47,48,49]. Removal of mannose residue i from the B-branch of the *N-*linked glycan (Figure 1) by α1,2-mannosidase, Mns1p in *S. cerevisiae* or ER α1,2-mannosidase I (ERManI) in higher eukaryotes [50,51], generates Man_8_GlcNAc_2_ isomer B (M8B) that represents a physiological step in the *N-*glycan processing (Figure 2). This mannose cleavage allows binding of different lectin sorting receptors, which regulate the export of native glycoproteins from the ER [52]. ERGIC-53 binds high-mannose-type oligosaccharides with broad specificity but interestingly this lectin does not discriminate between the monoglucosylated and deglucosylated oligosaccharides [53]. This broad sugar-binding specificity of ERGIC-53 may be advantageous for efficient removal of proteins from the ER upon ER stress [53]. Initially, it was thought that the trimming of only one specific mannose residue is sufficient for the generation of the ERAD targeting signal [6]. Recent evidence implicates the necessity of further trimming of at last one more mannose residue in yeast (mannose k, branch C) and possibly three to four α1,2-linked mannose residues in mammalian cells [54,55,56,57] (Figure 1). In contrast to glycans possessing eight mannose residues, the Man_6_GlcNAc_2_ (M6) or Man_5_GlcNAc_2_ (M5) species lack mannose residue g (Figure 1); therefore, they cannot be re-glucosylated by UGGT. In *S. cerevisiae* re-glucosylation seems not to be crucial, since they lack a functional homolog of UGGT [58], on the other hand *S. pombe* possesses this ER folding sensor, so these processes can vary among different yeast strains [36]. Both in yeasts and mammalian cells truncation of certain mannose residues creates the signal that is recognized by ERAD lectins. In *S. cerevisiae*, exposed α1,6-bonded mannose j becomes a ligand for lectin Yos9p that recruits misfolded glycoproteins and is required for degradation of multiple ERAD substrates [54,59,60,61]. In mammalian cells, ERAD lectins OS-9 and XTP3-B are recruited [62,63,64] (Figure 2). They primarily recognize α1,6-liked mannose j by the mannose-6-phosphate receptor homology (MRH) domain. OS-9 can also recognize α1,6-linked mannose e and c (Figure 1) [65]. Interestingly, the removal of mannose g is not a prerequisite for glycoprotein binding to OS-9 [63]. Considering the fact that the MRH domain of OS-9 recognizes N-glycans lacking the terminal mannose from the C branch and that the lectin activity of OS-9 is required for glycoprotein degradation [63], it can be concluded that ERAD targeting signal system can be partly conserved between yeast and humans.

The question that arises is whether the ERManI is able to catalyse extensive demannosylation of ERAD substrates. Results of recent experiments revealed that theoretically, under specific conditions it could be possible, however, upon ER stress, contribution of EDEM family proteins (see below) is necessary [66,67,68,69,70,71,72,73,74]. At physiological conditions, at very low basal concentration of ERManI [75] that could be dispersed in the rough ER or different ER-derived vesicles, removal of mannose i from branch B (Figure 1) represents the basic catalytic activity of ERManI, that at this stage acts equally well at both properly folded and misfolded substrates [47,76] (Figure 2). However, in the considerations about catalytic capabilities of ERManI the exact localization of ERManI and/or ERAD substrates as well as participation of other mannosidases are probably crucial. Initially, one of the hypothesis considered involvement of Golgi endomannosidases that are able to cleave mannoses from A branch of the oligosaccharide core [77]. In this hypothesis Golgi mannosidases should be temporary transported to the ER or alternatively ERAD substrates might be packed into vesicles and sorted to the Golgi for extensive mannose trimming. The second model appeared to be consistent with the observation that a number of ERAD substrates undergo vesicular cycling through early Golgi compartments [78,79,80]. Direct engagement of the Golgi complex in the ERAD process has been supported by a finding that describes contribution of three traditional mammalian Golgi-localized α-1,2-mannosidases (IA, IB, and IC) to the intracellular degradation of misfolded glycoproteins [81]. It was demonstrated that some terminally misfolded human α1-antitrypsin variant null (Hong Kong) (NHK) co-localizes with Golgi markers, suggesting that mannose trimming by Golgi α1,2-mannosidases can contribute to NHK degradation [81]. Surprisingly, the role of the Golgi complex in ERAD can also be attributed to ERManI activity. Human ERManI is a type II transmembrane protein that is predicted to function as an ER resident protein [82,83]. However, recently published results have demonstrated that ERManI predominantly resides in the Golgi complex, where it is subjected to *O*-glycosylation [84]. It has been suggested that the glycan-based tagging of substrates for ERManI takes place in the Golgi complex. Moreover, a direct interaction between ERManI and γ-COP, the gamma subunit of coat protein complex I (COPI) have been identified [85]. Both ERManI and γ-COPI were demonstrated to support efficient intracellular clearance of NHK what may suggest that ERManI contributes to the establishment of a multifunctional system that facilitates the retrieval of captured ERAD substrates back to the ER. These data provide a model for the spatial separation by which glycoprotein quality control components operate in mammalian cells. Another hypothesis for the physical dislocation of ERAD system components out of the rough ER assumes that specialized subregions of the ER exist [56,86], where the mannosidase concentration reaches much higher levels than in the rest of the ER. It was considered, that in these compartments the ERManI concentration might be similar to the *in vitro* experimental levels that cause extensive mannose removal [75,77,87]. It was recently demonstrated that ERManI can be sequestered, at the steady state, in dynamic ER-derived quality control vesicles (QCVs) that, upon ER stress, converge at the juxtanuclear ER-derived quality control compartments (ERQC) [88,89]. Interestingly, it has been shown that interactions between ERManI and glycoproteins take place in the QCVs what is due to the fact that several proteins undergo a folding cycle between the ER and QCVs [88,89]. Properly folded proteins are concentrated at ER exit sites for further export, whereas misfolded proteins are segregated at the ERQC [89]. It is considered that in QCVs and especially in ERQC high local concentrations of ERManI could trim all α1,2-linked mannose residues producing Man_5_GlcNAc_2_ [89]. 

This hypothesis is strongly supported by observations indicating that the glucosylation status of oligosaccharides is unimportant for ERManI reactivity [90], and by experiments showing that ERManI can recognize tertiary and/or quaternary structures of glycoproteins, trimming mannose residues at a faster rate from misfolded substrates [91]. All these results strongly suggest that enhanced exposure of the protein substrates to ERManI at the ERQC under ER stress should accelerate the trimming and delivery to ERAD [90]. However, it should be noted that ERManI expression is unaffected by ER stress [57], suggesting that its role is less important under conditions that *de facto* require extensive mannose trimming from misfolded glycoproteins. ERManI was designated as a “timer” that initiates the ERAD of newly synthesized glycoproteins unable to attain their native conformation [46], nevertheless studies conducted during last years revealed that ERManI (or Mns1p in yeasts) and/or Golgi mannosidases are not solely responsible for intensive protein demannosylation during ERAD. It appeared that ER proteins that are upregulated during ER stress, that can recognize ERAD substrates, and that play an important role in demannosylation of substrates are the chaperone family proteins EDEM [66,67,68,69,70,71,72,73,74].

### 3.2. Chaperone Lectins EDEM as Important ERAD Regulators

#### 3.2.1. General Characteristics of EDEM Family Proteins, Relationship between EDEM1 and ERManI, and ERAD Tuning

ER degradation-enhancing α-mannosidase-like proteins (EDEMs) comprising EDEM1, EDEM2 and EDEM3 belong to the glycosyl hydrolase 47 (GH47) family that also includes ERManI and the Golgi α1,2 mannosidases [92,93]. HA-tagged EDEM1 was initially identified in COS-7 cells as a type II transmembrane protein that is inserted into the membrane as a 69 kDa form, and which is then converted to a 78 kDa glycoprotein by core glycosylation at five Asn residues (Asn181, Asn198, Asn299, Asn342, Asn624) [66]. It was described that EDEM1 possesses an uncleaved signal sequence that serves as a transmembrane region binding to the membrane-associated region of calnexin and forming a functional complex [68]. However, further experiments, confirmed also by computational algorithms [70,94] revealed that EDEM1 is a luminal protein in HEK293 (Human embryonic kidney) cells. These apparently conflicting results might be explained by probable variations in signal peptide cleavage in different cell lines [93]. The topologies of EDEM2 and EDEM3 are not so controversial, both are soluble proteins of the ER [69,70,71]. EDEM2 is a 64 kDa glycoprotein with *N-*linked oligosaccharides at Asn90, Asn112, Asn289 and Asn450 [69,70]. EDEM3 (104 kDa) is glycosylated at Asn in positions: 118, 195, 504, 511, 810, 814 and 900 [71]. EDEM proteins are major targets of the ER-stress-induced Ire1/Xbp1 pathway [70,71,95] that functions as one of the regulatory parts of the unfolded protein response (UPR). Mammalian cells trigger UPR to increase their capacity for ERAD, which is easily saturated upon an increase in cargo load and/or accumulation of misfolded proteins in the ER [96,97,98]. Besides inositol-requiring ER to nucleus signal kinase-1 (IRE1), the UPR is signaled by two more transmembrane proteins with luminal domains that sense the changes in the ER environment: RNA-dependent protein kinase like ER kinase (PERK), and activating transcription factor-6 (ATF6) [99,100,101,102]. PERK is a serine/threonine kinase, IRE1 possesses both kinase and endoribonuclease domains [99,100,101,102]. 

All these sensors associate with the chaperone binding immunoglobulin protein (BiP, also known as GRP78) [103]. In the presence of unfolded proteins, BiP dissociates from the sensor molecules, allowing them to dimerize and become activated by auto-phosphorylation (PERK and IRE1) or become translocated to the Golgi and proteolytically cleaved (ATF6) [99,100,101,102,103]. PERK phosphorylates translation factor eIF2α what attenuates protein synthesis, limiting protein load. ATF6 promotes overexpression of many genes that encode ER-resident chaperones and folding assistants including: BiP, calnexin, calreticulin and PDI. IRE1 activates transcription factor XBP-1, which in turn induces expression of factors facilitating ERAD [99,100,101,102,103,104,105]. It has been demonstrated that overexpression of EDEM1, EDEM2 and EDEM3 accelerates release of terminally misfolded glycoproteins from the calnexin/calreticulin cycle thereby increasing their elimination from the ER lumen [66,67,68,69,70,71]. Regulation of the UPR response, including the relationship between UPR and ERAD, becomes a complicated issue. This relationship might be, at least partially, based on a functional partnership that exists between ERManI and EDEM1 to coordinate the enhancement of ERAD as a part of the mammalian UPR [106]. Initially, it has been proposed that the very low basal concentration of ERManI is an advantage to target terminally misfolded glycoproteins for ERAD [75]. In this model the timing of the glycan modification is relative to the prolonged conformation-based ER retention. It has been demonstrated that this low basal level of ERManI is controlled by lysosomal degradation of newly synthesized ERManI molecules by a mechanism that involves the amino-terminal cytoplasmic tail of the enzyme [107]. Thus, it was concluded that proteolytically driven checkpoint control of ERManI contributes to the establishment of a glycoprotein quality control step by which the efficiency of asparagine-linked glycoprotein conformational maturation is measured [107]. It is believed now that this system operates under basal conditions, however, during transcriptional elevation of EDEM1 the efficiency of glycoprotein ERAD is enhanced through the formation of a complex that suppresses the proteolytic downregulation of ERManI [106]. Thus, during ERAD, ERManI is stabilized as a downstream effector target of EDEM1.

The functional partnership that exists between ERManI and EDEM1 regulates the level of ERManI. Moreover, it seems that the concentration of EDEM1 in the ER is also strictly regulated and dependently on the cell status regarding ER stress, this protein can be dislocated to the appropriate ER-derived compartments. Under extensive production and accumulation of ERAD substrates, overproduced EDEM1, together with PERK and IRE1 is directed to the ERQC [62,89,108]. This enhances ERAD capacity when misfolded proteins accumulate [109]. On the other hand, at steady state, the level of EDEM1 similarly to ERManI have to be reduced. However, in contrast to ERManI, EDEM1 is separated from QCVs [89], but instead, this chaperone protein is localised into vesicles covered with LC3-I, named EDEMosomes [110,111]. They form cisternae that lack a recognizable COPII coat and that are larger than the vesicles involved in cargo protein transport from the ER to the Golgi [94]. It is assumed that EDEMosomes contain up to 80% of the cellular EDEM1 [94,109,110,112]. Although segregation of EDEM1 into these vesicles is followed by its rapid degradation by lysosomal enzymes, EDEMosomes are distinct from the LC3-II coated autophagosomes and according to this, inactivation of autophagy was reported not to be sufficient to prevent EDEM1 disposal [110]. This mechanism, defined as ERAD tuning ensures that under basal conditions, ERAD regulators are removed from the ER [110]. Otherwise, if present in excess, they could prematurely interrupt productive polypeptide folding. In contrast to these observations, it has been demonstrated that EDEM1 was observed in LC3A- and LAMP1-positive structures corresponding to autophagosomes [113] and that selective autophagy is involved in its degradation [114]. Despite the nature of vesicles involved in the EDEM1 disposal, it seems that degradation of this ER regulator occurs by a route differing from that followed by ERAD substrates [114,115], that are degraded by the 26S proteasome.

#### 3.2.2. Catalytic Activity of EDEM Proteins

All group members of GH47 family proteins share a similar single mannosidase homology domain, and all catalytic residues required for glycolytic activity and for binding of the specific inhibitor of α1,2-mannosidases kifunensine are conserved in the EDEM proteins [66,69,71,93,116,117]. Despite this homology, initial studies on yeast EDEM1 (Htm1p) [118] and the mammalian orthologs, EDEM1 and EDEM2, failed to reveal any hydrolytic activity [66,67,68,69,70,119]. However, further studies indicated that the EDEM orthologs in protists [120] and in yeasts [55,56,61] exhibit mannosidase activity, and that EDEM1 overexpressed in human cells accelerates demannosylation of terminally misfolded glycoproteins from branch A and C of *N-*glycans [72,73]. It has also been demonstrated that EDEM3 possesses α1,2-mannosidase activity *in vivo* [71], and that a modified EDEM1 and EDEM3 bearing a mutation in one catalytic residue conserved amongst α1,2-mannosidases (substitution E220Q and E147Q, respectively) failed to accelerate *N-*glycan disassembly [71,72,73]. This may suggest that both EDEM1 and EDEM3 are active mannosidases (Figure 2). EDEM2 α-mannosidase activity was the most controversial, however recently published results allow one to propose a completely novel mechanism involving a “double check” of glycosylated ERAD substrates in mammalian cells [74]. According to this model, mannose trimming from Man_9_GlcNAc_2_ to Man_8_GlcNAc_2_ form (removal of mannose i form B branch of the oligosaccharide core, Figure 1 and Figure 2) is conducted mainly by EDEM2, and downstream glycan transformation from Man_8_GlcNAc_2_ to Man_7_GlcNAc_2_ is performed mainly by EDEM3 and to a lesser extent by EDEM1 [74] (Figure 1 and Figure 2). It is assumed that extensive substrate demannosylation by EDEM proteins provides two signals that flag misfolded proteins for degradation [112]. Removal of mannose g (Figure 1) prevents restoring the monoglucosylated status of the glycan structure what irreversibly extracts ERAD candidates from the calnexin/calreticulin cycle. Cleave of mannose k, as was already mentioned, exposes α1,6-linked mannose j which becomes a target for crucial ERAD regulators [62,63,64,112] (Figure 1 and Figure 2). Mannose f can also finally be discarded [72,112]. Thus, EDEMs can control glycoprotein folding/misfolding status in mammalian cells in a very specific way, that helps to avoid unnecessary destruction of proteins still being in the folding cycle. This mechanism is much more complex than in yeast cells, where first mannose trimming is catalyzed by α-mannosidase, Mns1, and “single check”, performed by EDEM homolog (Htm1p) generates the *N-*glycan signal for glycoprotein degradation [54,55,61]. Despite all these advanced studies on EDEM proteins catalytic activity, demonstration of mannosidase activity with purified EDEM1, EDEM2 and EDEM3 is still lacking and these missing data have to be provided.

EDEM chaperone proteins accelerate substrate demannosylation, which unquestionably regulates ERAD. However this catalytic activity is not necessary for other functions of EDEMs. It has been demonstrated that EDEM1 inhibits formation of covalent aggregates upon release of misfolded proteins from the calnexin/calreticulin cycle, and this chaperone activity of EDEM1 is independent of substrate demannosylation [72,109,121,122]. The results of site-directed mutagenesis indicate that regulation of the level of ERManI does not require inherent mannosidase activity of EDEM1 [106]. Moreover, it has been demonstrated that substrate recognition by EDEM1 and EDEM2 might be glycan independent and thus not connected with catalytic activity of EDEM proteins [62,109,123,124,125,126,127,128,129,130,131].

#### 3.2.3. Interaction of EDEM Proteins with other ERAD Regulators

Glycan-Independent Interactions with early ERAD Pathway Regulators

One of the first developments concerning the role of EDEM proteins in ERAD, indicated interactions between EDEM1 and calnexin, but not between EDEM1 and calreticulin [68]. Since calnexin contains the transmembrane region that calreticulin lacks, it was suggested that this transmembrane region might be responsible for EDEM1 binding to calnexin [68]. EDEM2 also binds this lectin and the interactions are much stronger than interactions between EDEM1 and calnexin [131]. The EDEM3-calnexin association was observed to be negligible [131]. These interactions are not mediated through EDEMs glycan binding domains [131]. Accumulating evidence indicates that EDEM chaperone proteins may reside in complexes also with other ERAD regulators. It has been demonstrated that EDEM1 can functionally associate with a disulphide reductase ERdj5 that through its DnaJ domain interacts with the DnaJ-binding chaperone BiP (GRP78) [132]. ERdj5 possesses six thioredoxin-like domains, (four of which are active reductases and contain CXXC motifs) [133]). Due to its reductase activity and due to the interactions with BiP and EDEM1, it prevents the covalent multimer formation of misfolded proteins by disulphide bond cleavage [132]. In a proposed model, misfolded proteins are transferred from the calnexin/calreticulin cycle to EDEM1, then disulphide bonds of the ERAD substrate are cleaved by ERdj5, followed by interactions with BiP, a heat shock protein (Hsp)70 family molecular chaperone which conversion from ATP-form to ADP-form is generated by ERdj5 serving here as BiP cofactor [77,132]. This enables substrates to be strongly bound by BiP [132,134] and holds them in a dislocation-competent state until they are transferred to the retrotranslocation channel. It has been demonstrated that the *C*-terminal cluster of ERdj5, possessing reductase activity contains the site responsible for interaction with EDEM1 [135]. It is probable that EDEM1 preferentially recruits disulfide-linked dimers to ERdj5 in order to promote their reduction by the *C*-terminal part of ERdj5 [135]. The interaction between EDEM1 and Erdj5 is not due to the lectin property of EDEM1, since the *C*-terminal cluster of ERdj5 is not *N-*glycosylated. Interestingly, nonglycoprotein substrates are bound by BiP and then they are transferred to ERdj5 for disulfide bond cleavage, without passing the calnexin/EDEM1 pathway [136]. When glucose trimming of the *N-*glycan groups of the substrates is inhibited, glycoproteins are also targeted to the nonglycoprotein ERAD pathway. Thus, it is suggested that two distinct pathways for ERAD of glycosylated and non-glycosylated protein substrates operate in mammalian cells, and that these pathways are interchangeable under ER stress conditions [136].

Glycan-Dependent Interactions with late ERAD Pathway Regulators

Beside examples of glycan-independent interactions of EDEM1 with important ERAD regulators, lectin-carbohydrate interactions are crucial for EDEM1 association with the components of the ER dislocation machinery, especially SEL1L [124]. This protein is a part of the membrane-embedded HRD1-SEL1 ubiquitin ligase complex. OS-9 and XTP3-B that recognize misfolded substrates with oligosaccharides expressing extensive mannose trimming [62,63,64] associate with HRD1-SEL1, forming a complex including also BiP and GRP94 [137,138]. For EDEM1-SEL1L interactions the mannosidase-like domain of EDEM1 is necessary, on the other hand this domain does not appear to be required for ERAD substrate binding [124]. Thus, it was proposed that EDEM1 binds misfolded proteins and uses its mannosidase-like domain to target aberrant protein substrates to the HRD1-SEL1 complex [124]. Alternatively, EDEM1, OS9 and XTP3-B may each exist as oligomers, one subunit associating with the substrate and another with SEL1L [62]. The HRD1 complex, to which both OS9 and XTP3-B are associated, was shown to require oligomerization to be functional in yeast [62,139]. Moreover, it is possible that EDEM1 binding to SEL1L may involve bipartite interactions including its mannosidase-like domain recognizing the large and flexible glycans of SEL1L, and protein-protein association with the TPR (11 tetratricopeptide repeats) domains. Interactions with the TPR domains of SEL1L may be mediated directly through EDEM1 or a member of the EDEM1 complex such as BiP or ERdj5 [124]. Importantly and interestingly, signal sequence processing and co-translational glycosylation of EDEM1 are not effective processes. This results in heterogeneous glycosylation of EDEM1 creating a protein doublet, and additionally generates EDEM1 possessing dual topologies—both soluble and type II membrane protein [140]. It has been demonstrated that the membrane form of EDEM1 associates more efficiently with the membrane adapter SELlL, whereas soluble EDEM1 binds more effectively to soluble ERdj5 [140]. Thus, EDEM1 signal sequence processing can control its structure, localization, associations and function. Inefficient signal sequence cleavage is specific for mammalian EDEM1 among the EDEM-family members [69,70,71]. EDEM2 and EDEM3 associate with SELlL in their soluble forms [131,141]. EDEM2 binding to SEL1L is strong, comparable to EDEM1-SELlL and similarly to for EDEM1 these interactions are mediated via EDEM2 glycans [131]. However, not all EDEMs interact with SEL1L in the same way, since EDEM3 binding to this membrane receptor was found to be very weak [131]. This may indicate that EDEM3 operates in a manner significantly different from that of either EDEM1 or EDEM2.

Association of EDEM Proteins with the ER Retrotranslocation Machinery

There is not any reported evidence for direct interaction between any of the members of the EDEM family proteins and ER translocons—Sec61p or Derlin-1. Sec61p is the main translocon involved in co-translational protein transport into the ER [142,143] and also being part of the ERAD disposal machinery [144,145]. Derlin-1 was first demonstrated to be involved in the retrotranslocation of MHC class I heavy chains from the ER to the cytosol [146,147]. However, a more general role of this protein in the extraction of certain aberrantly folded proteins from the ER was also further confirmed [148,149,150]. It is believed that Derlin-1 forms a physical channel through which misfolded glycoproteins move from the ER to the cytosol [146,147], but it cannot be excluded that such channels are also formed by related proteins: Derlin-2 and Derlin-3. These are transmembrane proteins that span the ER membrane multiple times. They may form heterooligomers when expressed simultaneously and homooligomers when expressed singularily, additionally they are required for the ERAD of misfolded glycoproteins [151,152,153]. Derlin-1 can also form heterooligomers with Derlin-2 [151]. EDEM1 interacts with Derlin-2 and Derlin-3 [152]. Interestingly, overexpression of Derlin-2 was found to mediate the association of EDEM1 with a cytosolic AAA-ATPase p97. p97 couples ATP hydrolysis to the extraction of misfolded proteins from retrotranslocation sites and subsequently targets them for degradation. An immunofluorescence experiment indicated that Derlin-2 and Derlin-3 colocalise with Sec61β, a component of Sec61p translocon [152]. Thus, it is possible that EDEM1 can be a part of the large retrotranslocation complex, a structure that is directly or indirectly connected with Sec61p. There is not any evidence that EDEM1 interactions with Derlin-2, Derlin-3 or p97 are carbohydrate dependent.

#### 3.2.4. EDEMs Substrate Specificity

Model misfolded proteins became a powerful tool in the study of protein substrates recognition during ERAD. BACE457 and BACE476 are splice variants of human β-secretase, BACE501, lacking 44 and 25 amino acids (respectively), due to the in-frame deletions within their catalytic domain [67,154,155]. The majority of these proteins undergo inefficient folding in the calnexin cycle when they are transiently expressed in human cells. Extensively oxidized BACE457 was found in disulfide-bonded complexes associated with the luminal chaperone BiP and protein disulfide isomerase (PDI) [154]. Moreover, two human genetic variants of α1-antitrypsin (A1AT), null (Hong Kong) (NHK) [156] and PI Z [157] misfold in the ER and are subsequently degraded by the cytoplasmic proteasome [69,158]. Lack of A1AT in the serum is known to cause emphysema and/or liver cirrhosis [159]. EDEM1 overexpression resulted in faster release of membrane bound BACE457, the luminal form (BACE457Δ) and A1AT NHK from the calnexin/calreticulin cycle and earlier onset of their degradation [66,67,68,72,73], whereas EDEM1 downregulation led to the accumulation of misfolded proteins in the ER what finally delayed ERAD [67]. Interestingly, a combined effect of ERManI and EDEM1 on ERAD of misfolded A1AT was demonstrated [112]. Thus, a model assuming that the misfolded glycoproteins interact with ERManI and with EDEM1, before being recognized by downstream ERAD components was proposed [119]. EDEM2 and EDEM3 accelerate the disposal of BACE476 and A1AT, forms NHK and PI Z [69,70,71]. Results of the experiments performed with all these protein substrates indicated that lectin-carbohydrate interactions are important for substrate recognition by EDEMs. This observation was due to the facts that: (i) kifunensine which blocks trimming of α-1,2-mannose residues from *N*-glycans by ERManI and EDEMs [93,158] greatly inhibits degradation of misfolded protein in cells overexpressing EDEM1 and EDEM3, suggesting that EDEM1 and EDEM3-enhanced misfolded substrate disposal requires mannose trimming [66,67,71]; (ii) overexpression of the modified E147Q EDEM3 [71] and E220Q EDEM1 [72] that bear the mutations in one of the conserved acidic residues essential for enzyme activity of α1,2-mannosidases, abolished enhanced substrate demannosylation by EDEM1 and EDEM3 and finally; (iii) demonstration that all EDEM homologs possess α1,2-mannosidase activity *in vivo* [72,73,74]. However, other experiments revealed that EDEMs interactions with protein substrates are much more complex. Disruption of the EDEM1 mannosidase-like domain by introducing specific mutations showed that EDEM1 protein binding does not require the trimming of substrate glycans or even ERAD substrate glycosylation [124]. Similarly, EDEM1 binding to mutant P23H rod opsin (P23H opsin) was independent of mannose trimming [125]. It was demonstrated that association of EDEM1 with a well-studied model ERAD substrate, asialoglycoprotein receptor H2a does not require mannose trimming or ERManI [62,109]. Additionally, interactions of EDEM1, EDEM2 and EDEM3 with human sonic hedgehog (SHH) protein were also independent of the substrate glycosylation [131]. Thus, it was suggested that in addition to *N-*linked oligosaccharide moieties of glycoproteins, EDEM1 can recognize misfolded regions of aberrant proteins. This suggestion was in agreement with previously performed studies indicating that EDEM1 can directly interact with the protein toxin ricin [123]. This toxin has been widely used as a tool in cell biology studies and in medicine for a long time [160,161,162]. It also become very useful in the investigations of protein substrates recognition during ERAD [123,126,127,128]. Ricin holotoxin is a heterodimeric protein that consists of two polypeptide chains (A and B) joined by a disulfide bond. The ricin A-chain (RTA) inhibits protein synthesis by irreversibly inactivating eukaryotic ribosomes [163,164]. The B-chain (RTB) is a lectin which binds to β-1,4-linked galactose residues [165]. 

Inhibition of protein synthesis by RTA requires ricin retrograde transport from early endosomes to the Golgi complex and then to the ER. Translocation of RTA from the ER to the cytosol occurs after reduction of the internal disulfide bond present in holotoxin in a reaction that is catalyzed by the protein disulfide isomerase (PDI) [166,167]. Ricin A-chain is then directed to the cytosol through Sec61p ER translocation channel [123,168] in a similar way as misfolded proteins directed for ERAD. However, this toxin is not a typical ERAD substrate. It is not transported to the cytosol for protesomal degradation, instead ricin translocation out of the ER becomes a part of its intoxication route. EDEM2 directly promotes RTA transport to the cytosol [127], whereas, surprisingly, overexpression of EDEM1 decreases this transport [123,127]. High expression of both EDEM1 and EDEM2 increases extraction of misfolded proteins from the calnexin/calreticulin cycle [66,67,70] and boosts ERAD, thereby inhibiting access of ricin to the translocon [123]. However, in EDEM1-transfected cells treated with specific inhibitors (puromycin, kifunensine) that increase general accessibility of ER translocons, much more ricin can be transported to the cytosol in comparison to control cells. Additionally, both inhibitors significantly increase the interactions between ricin and EDEM1 [123]. Thus, it was concluded that EDEM1 promotes RTA transport to the cytosol but only when ER channels become more accessible for ricin [123]. On the other hand, increased accessibility of ER translocons, did not cause further enhancement of EDEM2-dependent retrotranslocation of RTA to the cytosol [127]. Thus, EDEM2-mediated retrotranslocation of RTA to the cytosol is not dependent on ER translocon accessibility. It has been demonstrated that EDEM1 and EDEM2 interact with ricin A-chain [123,126,127,128]. An important question concerns the nature of ricin interactions with EDEM1 and EDEM2. Ricin A-chain purified from plants contains two *N-*linked oligosaccharide chains [169]. However, toxin used in EDEM experiments is without *N-*glycans on the A-chain since recombinant RTA produced in *E. coli* lacks oligosaccharides. Thus, ricin can be considered as non-glycosylated ERAD substrate, confirming the significance of carbohydrate independent interactions with EDEM1 or EDEM2. Interestingly, the co-immunoprecipitation and “pull-down” experiments showed that more ricin can interact with EDEM2 than with EDEM1 [127]. 

Based on the data describing different translocon accessibility for ricin upon EDEM1 or EDEM2 overexpression, it is possible that EDEM2 recognizes ricin similarly to misfolded proteins, whereas EDEM1 has higher affinity to misfolded glycoproteins than to ricin. This is the first observation showing that EDEM1 and EDEM2 may differ in substrate specificity [127]. The differences between both lectin interactions with RTA and different role of EDEM1 and EDEM2 in ricin transport to the cytosol may be connected with dual topologies of EDEM1 and with probable differences in general substrate recognition by EDEM1 and EDEM2 [140]. EDEM1 is more effective at contribution in the disposal of substrates that possess similar topologies to this ER chaperone; the soluble form of EDEM1 was most effective at accelerating the turnover of the soluble ERAD substrates, whereas the membrane form of EDEM1 accelerated the degradation of a membrane-bound proteins [140]. These observations do not concern EDEM2, since this protein possesses homogenous, soluble form in all cells [69,70]. It has been demonstrated that EDEM1 uses its mannosidase homology domain to form complexes with components of the dislocation machinery (*i.e.*, SEL1L) rather than with misfolded proteins [124]. Moreover, it associates with a range of quality control factors, suggesting it can act in large protein complexes. It should be noted that ricin can utilize SEL1L to be translocated to the cytosol [170]. Other studies suggest that EDEM1 participates both in early and late steps of ERAD [109]. It is possible that EDEM1 is directly or indirectly involved in substrates mannose trimming and their direction to other components of ERAD machinery, while EDEM2, despite its probable role in initial demannosylation step [74], is involved in more direct recognition of misfolded proteins [127]. The differences in RTA recognition by EDEM1 and EDEM2 may also result from ERAD tuning [110]. Additionally, it is quite possible that different ERAD complexes are used to monitor different classes of misfolded substrates disposal. To support this hypothesis, it has recently been shown that different PDI family members play opposing roles during the degradation of an ERAD substrate in mammalian cells [171]. Thus, it cannot be excluded that under EDEM1 overproduction the translocon can be occupied by different types of substrates than during EDEM2 overproduction. This could also partially explain differences in ricin A-chain transport to the cytosol in EDEM1 and EDEM2 overexpressing cells.

It should be noted that in addition to ricin, mechanisms for ERAD targeting of several non-glycosylated proteins substrates were also analyzed. However, the role of EDEM1 in these processes is not completely clear, and in some cases seems to be contradictory. Degradation of three naturally non-glycosylated ERAD substrates: the unassembled non-secreted NS-1κ light chain (NS-1 κLC), a truncated Igγ heavy chain γ V-C_H_1, and a non-glycosylated mutant of H2a was dependent on EDEM1, suggesting shared ERAD pathway for glycosylated and non-glycosylated proteins [130]. It was demonstrated that EDEM1 associates with the non-glycosylated proteins through a region outside of its mannosidase-like domain [130]. Glycosylated, but also non-glycosylated mutants of tyrosinase, a tumor antigen overexpressed in melanoma cells, immunoprecipitated with EDEM1 even in the absence of its intact mannosidase-like domain [129]. Interestingly, for interactions with both types of substrates the *N*-terminal, intrinsically disordered (ID) region of EDEM1 was necessary [129]. On the other hand, EDEM1 did not bind or accelerate the turnover of two naturally non-glycosylated ERAD substrates: κ light chain and mutant transthyretin [124]. Moreover, EDEM1 was not involved in recruitment of mutated, non-glycosylated version of A1AT NHK to ERdj5 [136]. As was already mentioned in this review, Nagata and co-workers (2013) suggested occurrence of two distinct pathways for ERAD of glycoproteins and nonglycoproteins in mammalian cells. Interestingly, EDEM2 was required for ERAD of both glycosylated and non-glycosylated ERAD substrate sonic hedgehog (SHH), EDEM3 was only necessary for glycosylated SHH, and EDEM1 was dispensable for both [131].

**Figure 3:**
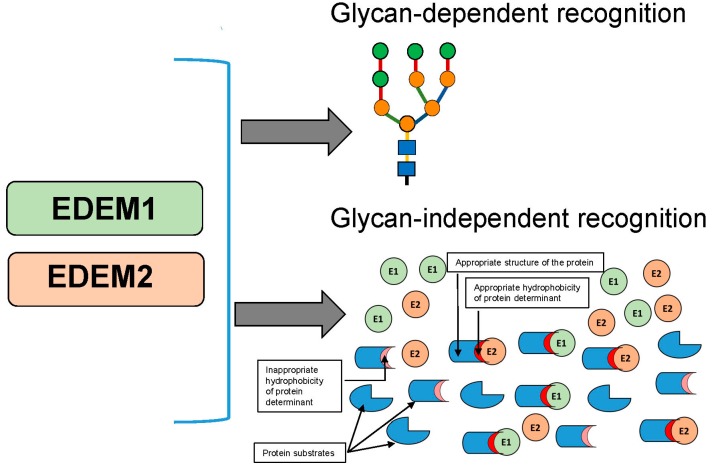
Glycan-dependent and glycan-independent interactions of EDEM1 and EDEM2 with protein substrates. For glycan-independent interactions with EDEM family chaperones both appropriate conformation and hydrophobicity of proteins seem to be important (see text). E1 is for EDEM1, E2 is for EDEM2.

Studies performed with ricin and model misfolded protein BACE457 undoubtedly demonstrated that hydrophobic regions of protein substrates are important determinants recognized by EDEM1 and EDEM2 [126,128]. Both ricin A-chain and BACE457 possess highly hydrophobic regions located at their C-terminal ends (Val245 to Val256 and Ile414-Val434, respectively) [126,128,172]. In ricin this region is hidden in the holotoxin, but upon A-chain and B-chain dissociation in the ER it becomes exposed. Substitution of proline into alanine at amino acid position 250 (P250A) results in a significant decrease in modified ricin cytotoxicity in Vero (African green monkey kidney) and in HEK293 cells as well as in reduced RTA_P250A_ retrotranslocation to the cytosol [126,127,172]. It also appeared that mutation P250A decreases the interaction between RTA and EDEM1 and between RTA and EDEM2 [126,127]. Importantly, this mutation changes RTA secondary structure. The CD spectrum of RTA_P250A_ indicated a higher amount of α-helices in comparison to wild-type RTA, what was concomitant with the decrease in β-sheets structures [126]. Thus, ricin A-chain recognition by EDEM1 and EDEM2 might be determined by the appropriate structure of the toxin (Figure 3). Interestingly, transport of modified RTA to the cytosol, in contrast to wild-type RTA, appears to be both EDEM1 [126] and EDEM2-independent [127]. This might be explained by reduced interactions of RTA with EDEM1 and EDEM2. Moreover, it has been demonstrated that at the physiologically relevant temperature of 37 °C the *C*-terminal hydrophobic region of RTA is exposed to the ER membrane interior prior to dislocation to the cytosol [173]. Insertion of the hydrophobic region into membranes results from changes in the secondary structure of RTA which loses some α-helical structures. It is possible that the P250A mutant possessing an elevated level of α-helices is unable to undergo additional conformational changes allowing it to be stably inserted into the ER membrane. EDEM proteins probably interact with RTA prior to membrane incorporation raising the question of whether these lectin chaperones can somehow contribute to RTA conformational changes before insertion into ER membrane.

However, not only the structure of protein substrates but also the degree of hydrophobicity of protein determinants might be important in interactions with EDEM1 and EDEM2 [128] (Figure 3). It has been demonstrated that reduced hydrophobicity of the RTA *C*-terminal region results in a significant decrease in RTA binding to EDEM1 and EDEM2. On the other hand, further increase in the hydrophobicity of this already highly hydrophobic region leave unchanged interactions between RTAand EDEM1 and between RTA and EDEM2 [128]. These results indicate that for interactions between both EDEM1 and RTA and EDEM2 and RTA appropriate hydrophobicity of the substrate is important; too low hydrophobicity of the *C*-terminal region of RTA results in impaired interactions with EDEM chaperone proteins. Similarly, interactions of both chaperone proteins with BACE457 possessing decreased hydrophobicity in its *C*-terminal region were significantly reduced. In other words, mutations responsible for decreased hydrophobicity of the *C*-terminal region of BACE457 influence its recognition by the chaperone proteins EDEM1 and EDEM2. Interestingly, this *C*-terminal region serves as transmembrane anchor. It can be concluded that: (i) EDEM proteins alone or as a part of larger complexes are able to recognize hydrophobic domains of misfolded ERAD substrates, both exposed and transmembrane; (ii) sufficiently high hydrophobicity of protein substrate determinants is important for interactions with EDEM1 and EDEM2; (iii) in addition to lectin-like activity, EDEM proteins may bind substrates similarly to classical chaperones (Figure 3). It has been demonstrated that EDEMs substrate recognition significantly contribute to its degradation [66,67,68,69,70,71,72,156]. In agreement with these observations, degradation of BACE457 with decreased hydrophobicity was significantly abolished [128]. EDEM1 can differentiate between proteins undergoing a folding process and terminally misfolded proteins directed for degradation [124]. This ability can also probably be attributed to EDEM2. The ability of EDEM1 and EDEM2 to recognize hydrophobic domains might represent an important step in this differentiation, likely based on more extensive exposure of hydrophobic domains by terminally misfolded glycoproteins.

Under debate is whether ubiquitinated membrane proteins are degraded by the proteasome *in situ* at the membrane or whether they are extracted prior to proteolysis. It has been demonstrated that several less-hydrophobic transmembrane sequences derived from multimeric transmembrane proteins can enter the ER lumen completely, where they are recognized by ER chaperone BiP (GRP78), which in turn directs the unassembled subunits for degradation [174]. Moreover, some integral membrane ERAD substrates, such as MHCI [175] and cystic fibrosis transmembrane conductance regulator (CFTR) [176], have been observed to reside in the cytoplasm when proteasome function is compromised. This mechanism suggests a necessity of transmembrane segments solubilisation prior ERAD. Indeed, it has been demonstrated that the transmembrane domain of the ERAD substrate, Ste6p, is released into the cytosol in a Cdc48/p97- and ATP-dependent manner [177]. The question that appears is whether the recognition of hydrophobic transmembrane domains by EDEM1 and EDEM2 is necessary for solubilization from the lipid bilayer or whether EDEMs recognize already solubilized transmembrane domains. Interactions between the hydrophobic transmembrane domain of BACE457 and EDEMs might explain differences between ERAD of membrane BACE457 and its luminal form BACE457∆. Degradation of BACE457∆ is faster than that of membrane BACE457; half-life of BACE457∆ is about 40 min, while that of BACE457 is 4 h [156]. Importantly, the lag phase for the soluble variant of BACE457 is only 15 min, whereas for membrane bound BACE457 it is 90 min [156]. These differences might be connected with a requirement for extraction of the transmembrane domain of BACE457 out of the ER membrane. It was suggested that EDEM1 serves as a quality control receptor that acts as a molecular link between misfolded proteins and SEL1L [124]. Recently published results show that degradation of BACE457 did not require SEL1L complex [178]. It is possible that the role of EDEM1 and EDEM2 in ERAD in some aspects is common for luminal and membrane substrates: extraction from the calnexin/calreticulin cycle and potential substrate demannosylation. However, after release from EDEM1 or/and EDEM2, terminal acceptors of misfolded membrane proteins might be different from luminal aberrant glycoproteins. As an example, it was demonstrated that membrane-bound H2a [62], and other membrane-bound ERAD substrates (TCRα, CD3δ) [179] are targets of not only the E3 ubiquitin ligase HRD1, but also other ligase complexes [62,179]. Moreover, disposal of soluble proteins possessing luminal lesions (ERAD-L(S) substrates) is strictly dependent on the HRD1, whereas ERAD of the membrane-tethered variants of the same folding-defective polypeptides remained unchanged upon inactivation of HRD1 [180]. Thus, binding of ERAD substrates to the membrane may change selection of their degradation pathway. Candidates for factors that could maintain the solubility of transmembrane domains include cytoplasmic chaperones (such as Cdc48p), proteasome associated factors such as Rad23p/Dsk2p [181], and the proteasomal 19S particle. EDEM1 and EDEM2 might significantly contribute to extraction of misfolded protein transmembrane domains out of the ER membrane. This new feature confirms the complex role of EDEM chaperone lectins in ERAD.

## 4. Concluding Remarks

Despite the huge progress in understanding of ERAD that has been made during the last decade, recognition and sorting to the ERAD pathway of misfolded proteins, both glycosylated and non-glycosylated as well as protein toxins are still not completely defined. EDEM chaperone proteins seem to play a central role in this complex process. However, accumulating evidence indicates that other ER-operating soluble and membrane-bond lectins are also important in ERAD regulation. Generally, it has been proposed that EDEMs participate in the direct or indirect removal of mannose residues generating demannosylated glycoproteins or can act as the receptors that recognize, bind and direct mannose-trimmed proteins for ERAD by extracting them from the calnexin/calreticulin cycle [14,66,67,68,69,70,71,72,73,74,77,93,124,182]. Currently, the ability of binding of *N-*glycans after mannose trimming is ascribed to the lectins OS9 and XTP3-B [62] (Figure 2). However, considering the fact that EDEM1 and EDEM2 can recognize hydrophobic regions of protein substrates [126,127,128], it is possible that these lectins can be involved in the initial detection of proteins that have to be degraded. EDEM2 and/or ERManI has been reported [74] to be involved in Man8B *N-*glycan generation of misfolded proteins, that are further demannosylated by EDEM1 and EDEM3 (Figure 2). If EDEMs can really act as mannosidases, EDEM1 and EDEM2 must behave in a nontraditional manner as they selectively and efficiently bind protein substrates, properties not shared by other glycosidases. Three models describing EDEMs role in the ERAD process can be discussed: (i) first model, in which recognition by EDEMs of ERAD substrates exposing their hydrophobic regions is necessary for substrate demannnosylation; (ii) second model, in which recognition of hydrophobic regions in the substrate and glycan demannosylation can be unrelated events and finally (iii) regardless of whether the first or second model is correct, EDEM1 and EDEM2 can probably actively deliver misfolded substrates to the retrotranslocating complex located at the ER membrane (Figure 2). It should be noted that it cannot be excluded that all three models can simultaneously operate for EDEMs recognizing different classes of substrates. To support this hypothesis, EDEM1, EDEM2 and EDEM3 can differ not only in α-mannosidase activity [74], but also in protein substrate specificity [127,128]. On the other hand, the topology of an ERAD substrate as well as its glycosylation status dictates the ERAD route or the machinery utilized for its turnover [124,180,183]. It is possible that EDEM1 and EDEM2, similarly to Derlin-1 functions at several steps in the ERAD pathway [62,184].

*N*-glycosylation is a unique, multidimensional process necessary for protein folding and ERAD regulation. *N*-glycans may play a role in organizing the folding process by promoting changes in backbone conformation in folding intermediates. Moreover, *N-*linked oligosaccharides are undoubtedly used as specific tags recognized by different chaperones involved in protein folding and degradation. On the other hand, lectin-carbohydrate interactions can stabilize various ER multi-molecular regulatory complexes. For these reasons it was proposed that oligosaccharides act both as ER folding and quality control flag signals and as docking sites that regulate the assembly and stability of the ERAD components [112].
